# Multi-omics segregate different transcriptomic impacts of anti-IL-17A blockade on type 17 T-cells and regulatory immune cells in psoriasis skin

**DOI:** 10.3389/fimmu.2023.1250504

**Published:** 2023-09-12

**Authors:** Jaehwan Kim, Jongmi Lee, Xuan Li, Norma Kunjravia, Darshna Rambhia, Inna Cueto, Katherine Kim, Vasuma Chaparala, Younhee Ko, Sandra Garcet, Wei Zhou, Junyue Cao, James G. Krueger

**Affiliations:** ^1^ Laboratory for Investigative Dermatology, The Rockefeller University, New York, NY, United States; ^2^ Dermatology Section, Veterans Affairs Northern California Health Care System, Mather, CA, United States; ^3^ Department of Dermatology, University of California, Davis, Sacramento, CA, United States; ^4^ Division of Biomedical Engineering, Hankuk University of Foreign Studies, Seoul, Republic of Korea; ^5^ Research Bioinformatics, Center for Clinical and Translational Science, The Rockefeller University, New York, NY, United States; ^6^ Laboratory of Single-cell Genomics and Population Dynamics, The Rockefeller University, New York, NY, United States

**Keywords:** psoriasis, multi-omics, single-cell RNA sequencing, IL-17, biologics impacts of IL-17A blockade on skin immune cell subsets

## Abstract

Durable psoriasis improvement has been reported in a subset of psoriasis patients after treatment withdrawal of biologics blocking IL-23/Type 17 T-cell (T17) autoimmune axis. However, it is not well understood if systemic blockade of the IL-23/T17 axis promotes immune tolerance in psoriasis skin. The purpose of the study was to find translational evidence that systemic IL-17A blockade promotes regulatory transcriptome modification in human psoriasis skin immune cell subsets. We analyzed human psoriasis lesional skin 6 mm punch biopsy tissues before and after systemic IL-17A blockade using the muti-genomics approach integrating immune cell-enriched scRNA-seq (n = 18), microarray (n = 61), and immunohistochemistry (n = 61) with repository normal control skin immune cell-enriched scRNA-seq (n = 10) and microarray (n = 8) data. For the T17 axis transcriptome, systemic IL-17A blockade depleted 100% of *IL17A* ^+^ T-cells and 95% of *IL17F* ^+^ T-cells in psoriasis skin. The expression of *IL23A* in DC subsets was also downregulated by IL-17A blockade. The expression of IL-17-driven inflammatory mediators (*IL36G*, *S100A8*, *DEFB4A*, and *DEFB4B*) in suprabasal keratinocytes was correlated with psoriasis severity and was downregulated by IL-17A blockade. For the regulatory DC transcriptome, the proportion of regulatory semimature DCs expressing regulatory DC markers of *BDCA-3* (*THBD*) and *DCIR* (*CLEC4A*) was increased in posttreatment psoriasis lesional skin compared to pretreatment psoriasis lesional skin. In addition, IL-17A blockade induced higher expression of *CD1C* and *CD14*, which are markers of CD1c^+^ CD14^+^ dendritic cell (DC) subset that suppresses antigen-specific T-cell responses, in posttreatment regulatory semimature DCs compared to pretreatment regulatory semimature DCs. In conclusion, systemic IL-17A inhibition not only blocks the entire IL-23/T17 cell axis but also promotes regulatory gene expression in regulatory DCs in human psoriasis skin.

## Introduction

Psoriasis is one of the most common organ-specific autoimmune diseases in the human population affecting 3.2% of the adult population (1–3). Psoriasis originates from the skin, but it progressively induces systemic inflammation that may accompany psoriatic arthritis, metabolic syndrome, diabetes, overt vascular inflammation, and cardiovascular disease (4–7). Increasing evidence indicates that the systemic impact of psoriasis shortens the lifespan of affected individuals by at least 3–5 years (8).

Psoriasis persists as a lifelong disease that rarely improves without treatment, but systemic administration of recent monoclonal antibodies targeting the IL-23/Type 17 T-cell (T17) autoimmune axis is highly effective for psoriasis treatment (3). In the current model of psoriasis immunopathogenesis, IL-23 from dendritic cells (DCs) triggers T-cells (T17 cells) to produce IL-17, and IL-17 induces inflammatory mediators in keratinocytes (KCs) such as IL-36γ. The inflammatory mediators from KCs amplify DC and T-cell recruitment and activation completing the feed-forward inflammatory loop.

Genomic medicine mapping of psoriasis-associated immune pathways in the human skin enabled the therapeutic success of IL-23/IL-17 antagonists. The high efficacy of most IL-23/IL-17 antagonists was identified through early phase IIb studies that incorporated total skin gene expression profiles on relatively small numbers of patients, often with <10 in a treatment cohort (9–11). Large phase II and III studies were needed mainly to develop safety profiles on a sufficient number of patients for new drug registration.

However, total skin gene expression profiling is confronting limitations to dissect the IL-23/T17 autoimmune axis for explaining recent clinical trial findings (1): systemic administration of an anti-IL-17A monoclonal antibody increased the expression of genes involved in skin homeostasis promotion and KC stem cell activation in psoriasis skin (10). (2) systemic administration of an anti-IL-17A or anti-IL-23p19 monoclonal antibody produced durable psoriasis improvement maintenance even after treatment withdrawal in a subset of psoriasis patients (9, 12–15). To understand immune tolerance promotion induced by IL-23/T17 autoimmune axis blockade, we need to segregate and compare the transcriptome of regulatory immune cell subsets in human psoriasis skin before and after blocking the IL-23/T17 autoimmune axis.

**Figure 1:**
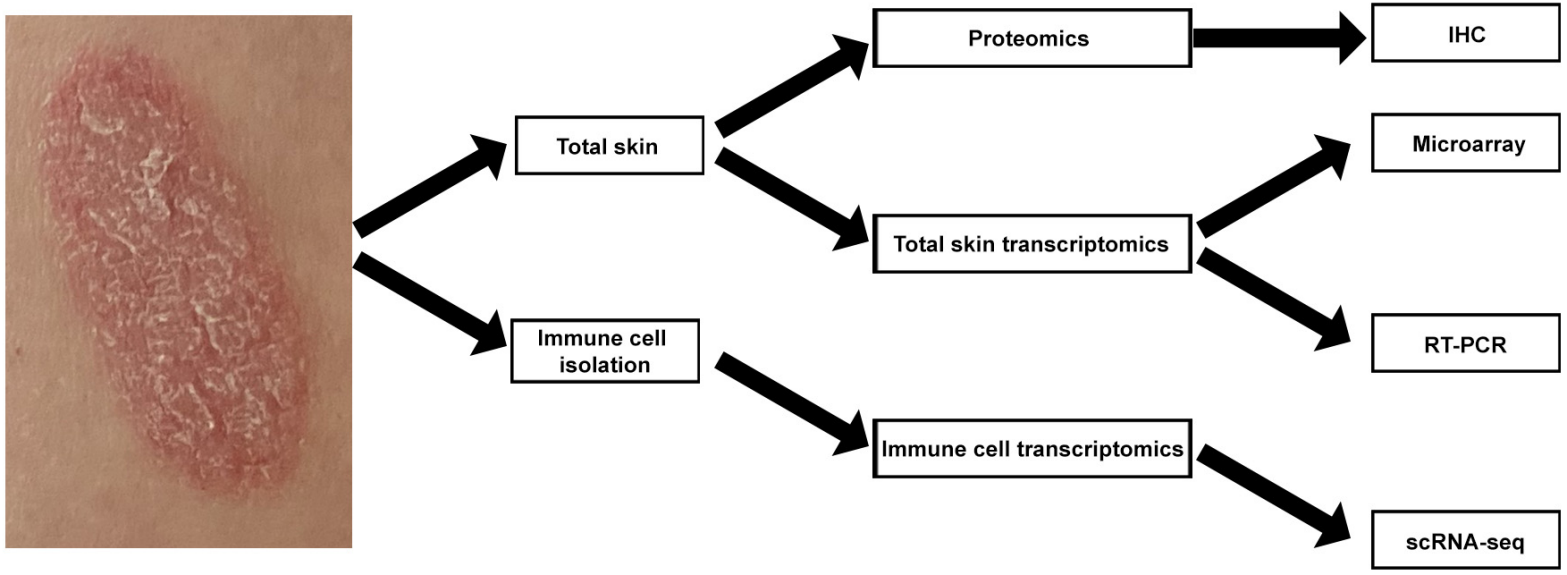
Schematic view of multi-omics strategy for mapping human psoriasis skin transcriptome.

We recently used RT-PCR analyses of total skin from a randomized placebo-controlled clinical trial (ClinicalTrial.gov identifier: NCT03131570) to study psoriasis skin transcriptome modifications induced by systemic IL-17A blockade (10). Here, we used archived samples from this trial for further analysis, applying a multi-omics approach. We performed single cell RNA-sequencing (scRNA-seq) analyses of T-cell, DC, and KC subsets isolated from psoriasis skin. We also used whole psoriasis skin for microarray, RT-PCR, and IHC analyses (**Figure 1**). Our multi-omics approach provides novel translational evidence in human psoriasis skin that systemic anti-IL-17A monoclonal antibody administration not only blocks the entire feed-forward inflammatory amplification loop between T-cells, DCs, and KCs, but also promotes regulatory transcriptome modification in regulatory DCs.

## Materials and methods

### Human psoriasis skin before and after systemic IL-17A blockade

23 adult psoriasis patients received anti-IL-17A monoclonal antibody (secukinumab) injections for more than 12 weeks in the phase II clinical trial (ClinicalTrial.gov identifier: NCT03131570). Patients received the anti-IL-17A monoclonal antibody at a dose of 300 mg with injections administered once weekly at baseline and at weeks 1, 2, 3, and 4 and then every 4 weeks. The clinical responses of patients and RT-PCR analyses of psoriasis skin were previously published (10).

For this study, psoriasis skin biopsy samples obtained from the clinical trial (10) were further analyzed by immune cell-enriched scRNA-seq (n = 18; GSE183047), microarray (n = 61; GSE226244), and immunohistochemistry (n = 61) together with our repository normal control skin immune cell-enriched scRNA-seq data (n = 10) (16) and microarray data (n = 8) (**Supplementary Table 1**). To minimize the batch effects, all the molecular experiments from the initial sample processing to cDNA library construction were performed by the same investigators under the same protocol.

Lesional biopsy specimens were harvested with a 6 mm punch biopsy from areas with representative psoriasis lesions. The individual skin sample information, including age, gender, psoriasis versus (*vs*.) control, and timepoint of biopsy is summarized in **Supplementary Table 1**. Psoriasis lesional skin biopsy samples before anti-IL-17A monoclonal antibody injections (pretreatment) and psoriasis lesional skin biopsy samples after 12 weeks of anti-IL-17 monoclonal antibody injections (posttreatment) were compared for this study.

Harvested skin samples were bisected immediately after the skin biopsy. Half of the two bisected skin tissue was incubated in a culture medium for single-cell RNA sequencing (scRNA-seq) experiments. Another half of the bisected skin tissue was snap-frozen and embedded in the Optimal Cutting Temperature (OCT) compound (Ted Pella, Redding, CA) for microarray and immunohistochemistry experiments.

### Immune cell-enriched single-cell RNA sequencing analyses of human psoriasis skin

#### Harvesting emigrating cells from skin biopsy tissues for immune cell-enriched scRNA-seq

Skin biopsy samples were incubated in culture medium for harvesting emigrating cells (16). To split the epidermis and dermis where most of the inflammatory cells were located, harvested skin tissue was immediately placed in 0.2% Dispase II (Sigma-Aldrich, St. Louis, MO) and incubated in a humidified incubator at 37°C and 5% CO_2_ for 3 hours. Then, the epidermis and dermis were separated with forceps and sliced into small pieces with #10 blade scalpels. The epidermis and dermis were separately incubated in RPMI-1640 medium with L-glutamine (Cytiva, Marlborough, MA) supplemented with 10% human albumin serum (Sigma-Aldrich, St. Louis, MO) in a humidified incubator at 37°C and 5% CO_2_. Nonplastic adherent cells that had emigrated out of the epidermis and dermis were harvested after 48 hours. The harvested cells from the epidermis and dermis were filtered through a 40-µm cell strainer (Corning, Glendale, AZ) and stored on ice. The cell numbers and viability were determined using a Countess automated cell counter (Invitrogen, Carlsbad, CA) and trypan blue staining (BioRad, Hercules, CA).

#### Single-cell capture and cDNA library preparation

The 10x Genomics Chromium Single Cell 3′ Reagents Kit user guide (https://support.10xgenomics.com) was used to prepare the single-cell suspension. The appropriate volume of each sample was diluted to recover 10,000 cells. Subsequently, the single-cell suspension, gel beads, and oils were added to the 10X Genomics single-cell chip. After droplet generation, samples were transferred into PCR tubes and we performed reverse transcription using a ProFlex PCR Thermocycler System (Applied Biosystems, Foster City, CA). After reverse transcription, cDNA was recovered using a recovery agent, provided by 10X Genomics, followed by silane DynaBead clean-up as outlined in the user guide.

#### Single-cell RNA sequencing data generation

The raw sequencing data for individual samples were processed using the 10X Genomics standard sequencing protocol without modifications. The FASTQ files were aligned to the human genome reference sequence GRCh38. Cell Ranger was applied to FASTQ files to generate files containing a barcode table, a gene table, and a gene expression matrix. The number of reads, mean reads per cell, valid barcodes, sequencing saturation, and other scRNA-seq parameters are detailed in **Supplementary Table 1**. The scRNA-seq data have been deposited in NCBI’s Gene Expression Omnibus and are publicly accessible through GEO Series accession number GSE183047. There is no restriction on the use of the data.

#### Single-cell data quality control

We used the *Seurat* R package (version 4.0) installed in R (version 4.0.2) for the downstream single-cell analyses (17–19). Before data integration, single-cell data quality control was performed separately for each sample as previously described for human skin single-cell RNA sequencing analyses (16, 20–22). Genes expressed in <3 cells, and cells with <100 or >5,000 genes and a mitochondrial gene percentage of >25% were filtered out to eliminate partial cells and doublets. Ubiquitously expressed ribosomal protein-coding (RPS and RPL) and MALAT noncoding RNA, miRNA, and snoRNA genes were excluded from the analysis as we described in our prior single-cell analysis publication with the publicly available R scripts (16). Seurat objects were created followed by normalizing data, scaling data, and finding variable 2,000 genes.

#### Single-cell data integration and harmonization

We first merged single-cell data of samples with the identical reagent kit version and the identical sequencer. To harmonize merged groups into a single dataset reducing batch effects, correspondences between cells in merged datasets were identified by the FindIntegrationAnchors function, and the correspondences were used for data integration with the IntegratedData function as detailed by Stuart et al. (18).

#### Non-linear dimension reduction and clustering analysis

Principal component analysis and graph-based clustering analysis were performed, and sixty principal components (PCs) were selected for Uniform Manifold Approximation and Projection for Dimension Reduction. With a resolution of 0.8, cells were clustered by the FindClusters function. The average gene expression of psoriasis *vs*. control cells within a cluster was calculated by the AverageExpression function.

When 10,000 cells are submitted to a microfluidic platform for droplet-based single-cell library construction, 2.3% to 4.6% of single-cell data could be technical artifacts caused by cell doublets formed during cell capture (Chromium Next GEM Single Cell 3′ Reagent Kits User Guide, 10X Genomics, USA). To remove clusters with possible doublets for downstream single-cell analysis, we excluded clusters expressing gene signatures of more than two different immune cell types (**Supplementary Figure 1**). We also excluded clusters of a minimal number of cells that were spatially separated from the analogous type of cells. To compare psoriasis and control cells within clusters representing each type of skin immune cells, we merged adjacent clusters of common immune cell subsets.

### Microarray analyses of human skin

Skin biopsy samples were frozen and then mechanically disaggregated before RNA extraction. RNA was extracted with RNA isolation kits (Qiagen) according to the manufacturer’s protocol. A total of 100 ng of biotinylated cDNA was hybridized to the GeneChip Human Genome U133 Plus 2.0 Array (Affymetrix). The expression values were obtained using the GCRMA algorithm (23), while normalization across samples was carried out using quantile normalization. The raw microarray data have been deposited in NCBI’s Gene Expression Omnibus (GEO) and are accessible through accession number GSE226244.

### RT-PCR analyses of human skin

Total RNA was extracted from frozen skin biopsies by the Qiagen miNeasy Mini Kit (Valencia, CA, USA). DNA was removed with on-column DNase digestion by the Qiagen RNase-free DNase Set (Valencia, CA, USA). The quality of extracted RNA was examined using Agilent Bioanalyzer 2100 (Agilent Technologies, Palo Alto, CA). Real-time PCR was performed on QuantStudio 7 Flex Real-Time PCR System (Thermo Fisher Scientific, Waltham, MA) with TaqMan Array Cards (384-well plates preloaded with TaqMan assays, Thermo Fisher Scientific, Waltham, MA). All primers are listed in **Supplementary Table 2**.

### Immunohistochemistry analyses of human skin

For immunostaining, vertical sections were cut to a thickness of 6 μm (+/- 1 μm) from the frozen skin biopsy tissues embedded in the OCT compound. Frozen sections were dried at room temperature and then fixed for 2 minutes in acetone. Next, the samples were blocked with 10% normal serum of the species in which the secondary antibody was made, and then the samples were incubated overnight at 4°C with the appropriate primary antibody. Primary antibodies used in this study were all mouse antibodies: KRT16 (LSBio, Seattle, WA; clone 7A4, dilution 1:500), CD3 (BD Biosciences, San Jose, CA; clone SK7, dilution 1:100), and CD11c (BD Biosciences, San Jose, CA; clone Bly6, dilution 1:100). Biotin-labeled horse anti-mouse antibodies (Vector Laboratories, Burlingame, CA) were used to detect the primary antibodies. The staining signal was amplified with avidin-biotin complex (Vector Laboratories, Burlingame, CA) and developed using chromogen 3-amino-9-ethyl carbazole (Sigma-Aldrich, St. Louis, MO). Section images were acquired at ×10 magnification. The number of immunostaining-positive cell counts per section was manually counted using computer-assisted image analysis software (ImageJ V1.48, National Institute of Health, Bethesda, MD). 61 sections were quantified for CD3 immunostaining (n = 40 for pretreatment sections and n = 21 for posttreatment sections) and 60 sections were quantified for CD11c immunostaining (n = 40 for pretreatment sections and n = 20 for posttreatment sections).

### Statistics

Statistical analyses of scRNA-seq data at the level of total T-cells, DCs, or KCs were performed under the general framework of the Seurat R package (17–19). We used a Wilcoxon rank sum test to define differentially expressed genes. A value of *p* < 0.05 was considered statistically significant.

Statistical analyses of scRNA-seq data at the level of T-cell and KC subsets were performed under the trajectory inference framework of Monocle3 R package (24, 25). We used a spatial autocorrelation analysis called Moran’s I (25) with the graph-test function, which Cao et al. (24) showed to be effective in finding genes that are differentially expressed in single-cell RNA sequencing data. A *p*-value that has been adjusted for the False Discovery rate < 0.05 was considered statistically significant (**Supplementary Tables 3**, **4**). Spearman’s rank correlation coefficient was calculated to test inter-correlations between the single-cell data.

For statistical analyses of microarray data, log2-transformed expression values were modeled using linear mixed-effects models. *P*-values from moderated t-tests were adjusted for multiple hypotheses across genes using the Benjamini-Hochberg procedure. A value of *p* < 0.05 and a fold change ratio > 2 was considered statistically significant.

For statistical analyses of RT-PCR data, RT-PCR results were modeled with the delta Ct (ΔΔCt) method for relative quantification to a housekeeping gene *RPLP0* (Ribosomal Protein Lateral Stalk Subunit P0) and calibration with a reference sample Control01 under the general framework of *pcr_analyze* R package (26) as we described in our prior publication (10). Two-sided Wilcoxon rank-sum test was used to compare RT-PCR experiment results between group 1 and group 2. A value of *p* < 0.05 was considered statistically significant.

For statistical analyses of immunohistochemistry data, we used a Wilcoxon rank sum test to compare measures between groups. A value of *p* < 0.05 was considered statistically significant.

### Study approval

The study protocol and informed consent were approved by the Institutional Review Board of the Rockefeller University, New York, NY, USA. The study was conducted in accordance with Good Clinical Practice and the Declaration of Helsinki, and all subjects provided written informed consent before entering the study.

## Results

### IL-17A blockade reduces T-cell signatures, dendritic cell signatures, and keratinocyte hyperproliferation signatures and increases regulatory molecular expression in psoriasis lesional skin at total skin transcriptome levels

**Figure 2:**
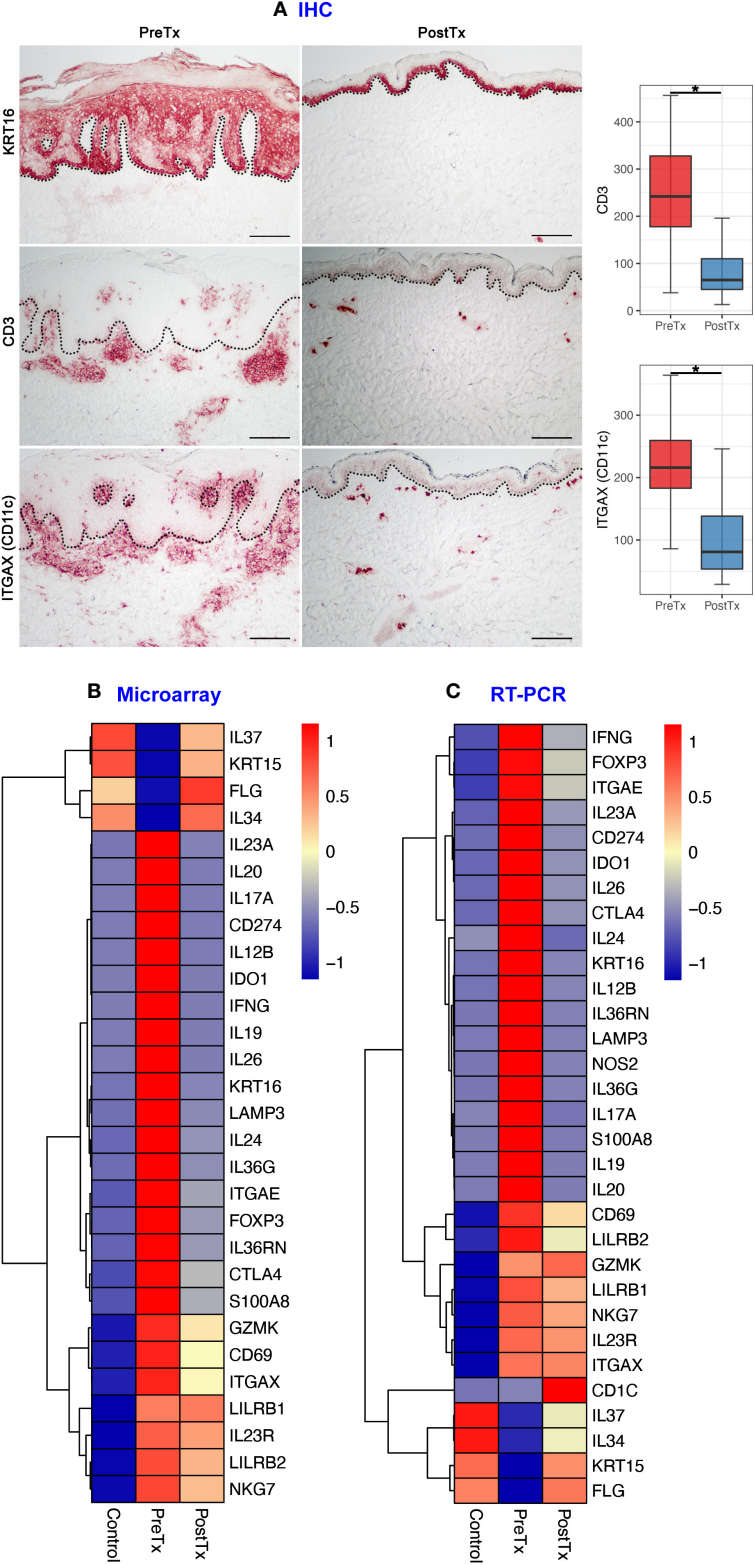
IL-17A blockade reduces T-cell signatures, dendritic cell signatures, and keratinocyte hyperproliferation signatures and increases regulatory molecular expression in psoriasis lesional skin at total skin transcriptome levels. **(A)** Immunohistochemistry of keratinocyte hyperproliferation (KRT16), CD3^+^ T-cells, and ITGAX (CD11c)^+^ dendritic cells (DCs) before (PreTx) and after (PostTx) 12 weeks of systemic anti-IL-17A monoclonal antibody (secukinumab) administration. Bar graphs display CD3^+^ T-cell (n = 40 for PreTx and n = 21 for PostTx) and ITGAX (CD11c)^+^ DC (n = 40 for PreTx and n = 20 for PostTx) number changes induced by systemic anti-IL-17A blockade. *p < 0.05. Black dotted lines delineate the junction between the epidermis and dermis (Scale bar = 200 μm). **(B, C)**, Differentially expressed genes (DEGs) between PreTx and PostTx at total skin transcriptome levels. DEGs that are relevant to psoriasis pathogenesis and statistically significant in both microarray (B, fold change ratio > 2 and *p* < 0.05) and RT-PCR (C, *p* < 0.05) experiments are displayed.

We first studied how the immune cell numbers are changed in psoriasis skin after systemic IL-17A blockade, and how the immune cell number changes are reflected in total skin transcriptome. KC hyperproliferation [KRT16 staining (27)], T-cell numbers (CD3 staining), and DC numbers (ITGAX (CD11c) staining) were decreased in psoriasis lesional skin after 12 weeks of anti-IL-17A monoclonal antibody injections (posttreatment) compared to pretreatment psoriasis lesional skin of the same patients (**Figure 2A**, *p* < 0.05).

Reflecting the decreased T-cell numbers by systemic IL-17A blockade, total skin transcriptome of T17 responses (*IL17A*, *IL26*, *IL12B*, and *IL20*) (3, 28), T-cell regulation (*IL24* and *CTLA4*) (29, 30), resident memory T-cells (Trms) [*ITGAE* (*CD103*)] (31) and regulatory T-cells (Tregs) (*FOXP3*) (32) was decreased in posttreatment psoriasis lesional skin compared to pretreatment psoriasis lesional skin in both microarray and RT-PCR experiments (**Figures 2B, C**, *p* < 0.05).

Reflecting the decreased DC numbers by systemic IL-17A blockade, total skin transcriptome of T17 cell-stimulating DCs (*IL23A*) (3) and mature DCs (*LAMP3* (*DC-LAMP*), *CD274* (*PD-L1*) and *IDO1*) (16, 33, 34) were decreased in posttreatment psoriasis lesional skin compared to pretreatment psoriasis lesional skin in both microarray and RT-PCR experiments (**Figures 2B, C**, *p* < 0.05).

Reflecting the decreased KC hyperproliferation by systemic IL-17A blockade, total skin transcriptome of KC cytokines that stimulate DCs and T-cells in feed-forward mechanisms (*S100A8* and *IL36G*) (3) was decreased in posttreatment psoriasis lesional skin compared to pretreatment psoriasis lesional skin in both microarray and RT-PCR experiments (**Figures 2B, C**, *p* < 0.05).

In contrast, total skin transcriptome of Treg-specific cytokine that mediates immune tolerance [*IL34* (35–37)], an anti-inflammatory cytokine that inhibits innate immune signaling [*IL37* (38, 39)], and KC stem cell marker of quiescence [*KRT15* (40)] were increased in posttreatment psoriasis lesional skin compared to pretreatment psoriasis lesional skin in both microarray and RT-PCR experiments, albeit the decreased T-cell, DC, and KC numbers (**Figures 2B, C**, *p* < 0.05).

### IL-17A blockade reduces type 17 T-cell signatures in psoriasis lesional skin at single-cell cluster levels.

**Figure 3:**
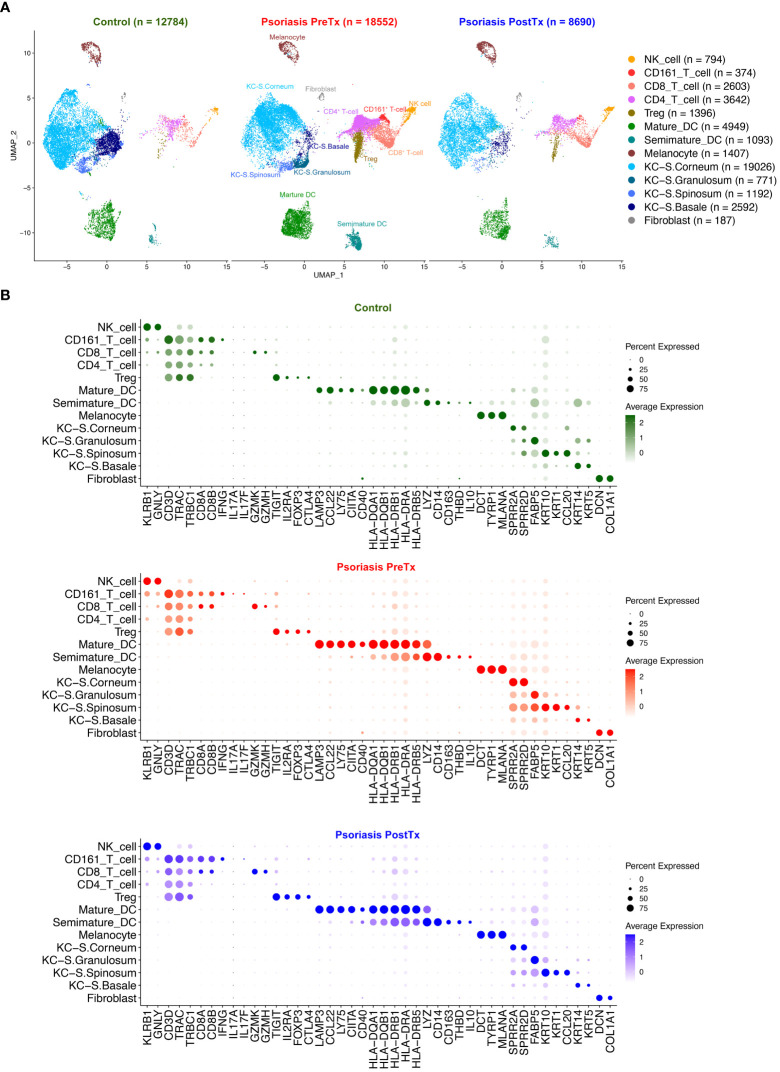
Human skin immune cell subset single-cell transcriptome comparison between psoriasis pretreatment (PreTx), psoriasis posttreatment (PostTx), and control (healthy volunteers). Psoriasis posttreatment is after anti-IL-17A monoclonal antibody (secukinumab) subcutaneous injections at a dose of 300 mg at baseline and weeks 1, 2, 3, 4, 8, and 12 (12 weeks of injections). **(A)** The Uniform Manifold Approximation and Projection plot (UMAP). **(B)** Dot plots displaying expression levels of cluster-defining genes.

**Figure 4:**
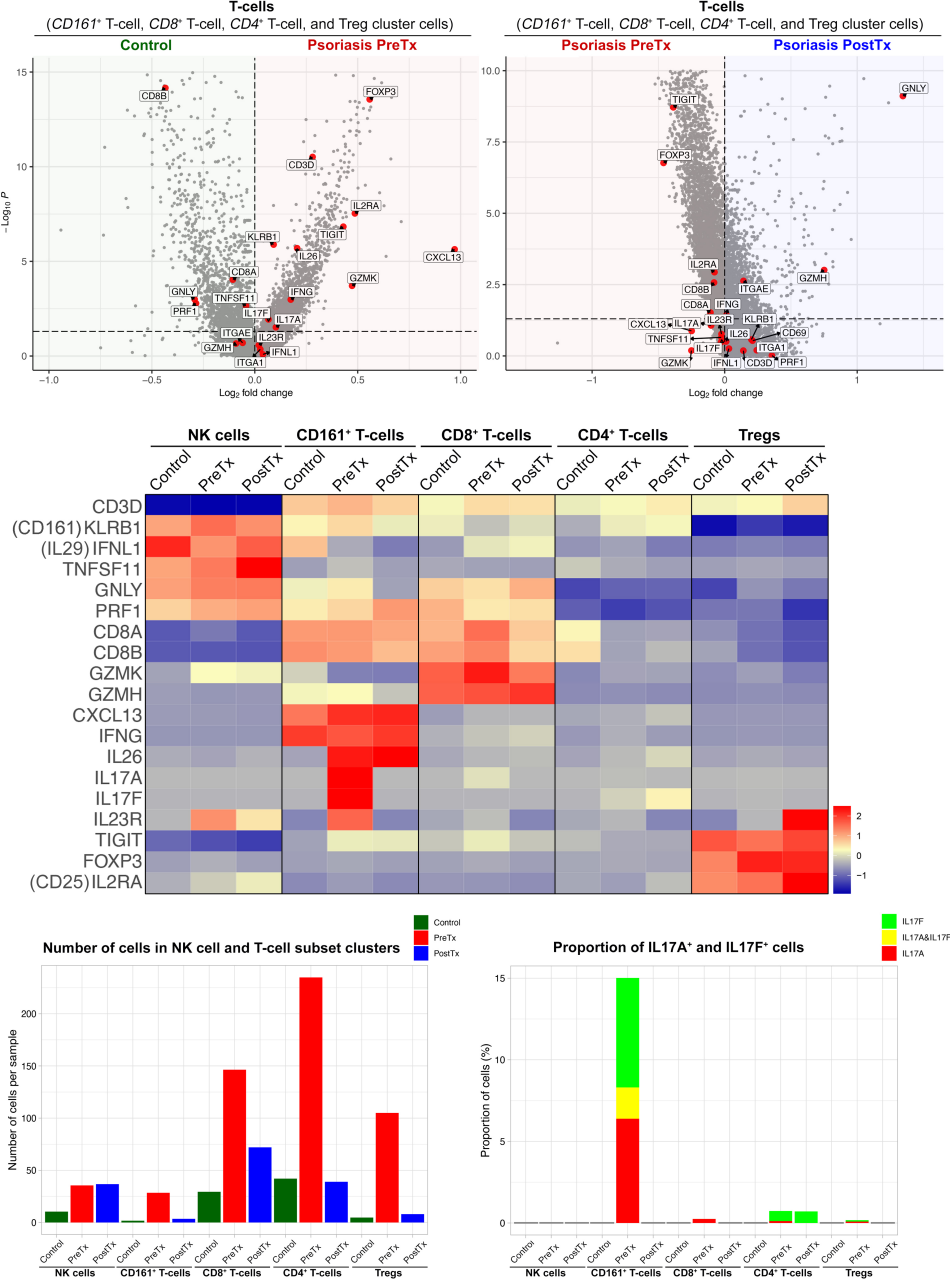
IL-17A blockade reduces Type 17 T-cell signatures and modifies regulatory T-cell signatures in psoriasis lesional skin at single-cell cluster levels. **(A)** Volcano plots displaying differentially expressed genes between control skin T-cells, psoriasis lesional skin T-cells before treatment (PreTx), and psoriasis lesional skin T-cells after 12 weeks of systemic anti-IL-17A monoclonal antibody (secukinumab) administration (PostTx). **(B)** Heatmap illustrating the average gene expression in clusters of NK cell and T-cell subsets, split by psoriasis PreTx, psoriasis PostTx, and control skin. **(C)** (Left) Number of cells per sample and (Right) proportion of *IL17A* ^+^ and *IL17F* ^+^ cells in NK cell and T-cell subset clusters, split by control, psoriasis PreTx, and psoriasis PostTx.

We next studied how systemic IL-17A blockade modified transcriptome of T17 cells and transcriptome of Tregs differently with single-cell analyses. Clustering analysis of 40,026 single cells from 28 samples of 20 subjects identified clusters of NK cells, CD161^+^ T-cells, CD8^+^ T-cells, CD4^+^ T-cells, Tregs, mature DCs, semimature DCs, melanocytes, KCs in different layers of Stratum (S.) corneum, S. granulosum, S. spinosum, S. basale, endothelial cells, and fibroblasts without subclustering (**Figure 3A** and **Supplementary Figure 2**). The identity of each cluster was determined by the expression of established cell type markers (**Figure 3B**). To characterize T17 cell transcriptome in psoriasis skin, we segregated T-cell (CD161^+^ T-cell, CD8^+^ T-cell, CD4^+^ T-cell, and Treg) clusters in the scRNA-seq data and tested if psoriasis T-cells express more T17 cell genes than control T-cells. The expression of T17 cell genes [*KLRB1* (*CD161*) (41), *IL17A*, *IL17F*, and *IL26* (42, 43)], a cytokine that represents a particularly T17-specific abnormality and positively associates with psoriasis severity (*CXCL13*) (44–47), and Treg genes (*FOXP3*, *IL2RA* (*CD25*), and *TIGIT*) was increased in pretreatment psoriasis lesional skin T-cells compared to control skin T-cells (**Figure 4A**, *p* < 0.05).

When we defined T17 cells as T-cell cluster cells expressing *IL17A* and/or *IL17F*, the majority of T17 cells were within the CD161^+^ T-cell cluster. 5.5% of total T-cells were CD161^+^ T-cell cluster cells and 15.0% of CD161^+^ T-cell cluster cells were T17 cells (**Figure 4C**). 76.5% of *IL17A* ^+^ T-cells, 61.4% of *IL17F* ^+^ T-cells, and 100% of *IL17A* ^+^ *IL17F* ^+^ T-cells were within the CD161^+^ T-cell cluster in pretreatment psoriasis lesional skin. In addition, 84.7% of CD161^+^ T-cell cluster cells expressed *CXCL13*, while only 1.6% of CD8^+^ T-cell cluster cells, 2.5% of CD4^+^ T-cell cluster cells, and 0.6% of Treg cluster cells expressed *CXCL13* in pretreatment psoriasis lesional skin (**Figure 4B**).

After systemic IL-17A blockade (12 weeks of anti-IL-17A monoclonal antibody injections), the expression of *IL17A* in the CD161^+^ T-cell cluster was decreased to zero in psoriasis lesional skin (**Figures 4B**, **C**, and **Supplementary Table 3**; *p* < 0.05). Although anti-IL-17A monoclonal antibody does not directly target IL-17F, the expression of *IL17F* was also decreased in the CD161^+^ T-cell cluster in psoriasis lesional skin after IL-17A blockade (**Figure 4B** and **Supplementary Table 3**, *p* < 0.05). When all T-cell cluster cells were considered, IL-17A blockade decreased 95% of *IL17F* ^+^ T-cells (**Figure 4C**).

### IL-17A blockade increases regulatory dendritic cell signatures in psoriasis lesional skin at single-cell cluster levels

**Figure 5:**
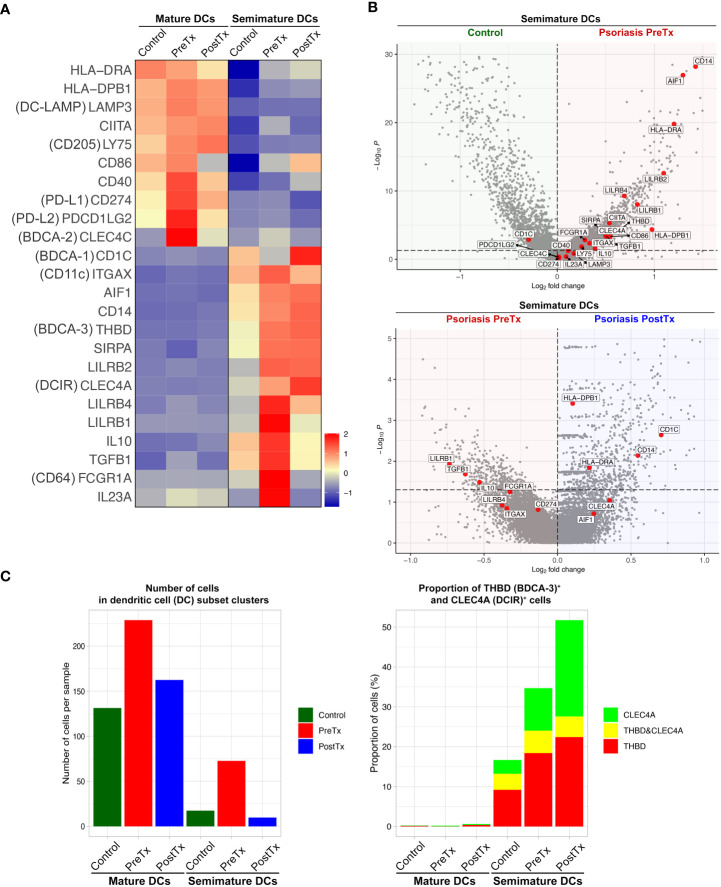
IL-17A blockade increases regulatory dendritic cell signatures in psoriasis lesional skin at single-cell cluster levels. **(A)** Heatmap illustrating the average gene expression in clusters of mature DCs and semimature DCs, split by control skin DCs, psoriasis lesional skin DCs before treatment (PreTx), and psoriasis lesional skin DCs after 12 weeks of systemic anti-IL-17A monoclonal antibody (secukinumab) administration (PostTx). **(B)** Volcano plots displaying differentially expressed genes between control skin DCs, psoriasis PreTx DCs, and psoriasis PostTx DCs. **(C)** (Left) Number of cells per sample and (Right) proportion of *THBD* (*BDCA-3*)^+^ and *CLEC4A* (*DCIR*)^+^ cells in DC subset clusters, split by control, psoriasis PreTx, and psoriasis PostTx.

We next studied how systemic IL-17A blockade modified transcriptome of different DC subsets that we previously described in our prior single-cell studies of human psoriasis skin (16). When we segregated DC subset (mature DC and semimature DC) clusters in the scRNA-seq data and compared their transcriptome, mature DCs in psoriasis skin were characterized by relatively high expression of MHC class II molecules (*HLA-DRA* and *HLA-DPB1*) and upregulated maturation markers [*CD86* (48) and *DC-LAMP* (*LAMP3*) (49)] (16) (**Figure 5A**). In contrast, semimature DCs in psoriasis skin were characterized by relatively low expression of MHC class II molecules (*HLA-DRA* and *HLA-DPB1*) and upregulated inflammatory monocyte receptor markers [*CD14* and *CD64* (*FCGR1A*) (50)] (**Figure 5B**, *p* < 0.05). Semimature DCs were characterized as regulatory DCs expressing high levels of *IL10*, *THBD* (*BDCA-3*), *LILRB2*, and *CLEC4A* (*DCIR*) (51, 52) (**Figure 5B**, *p* < 0.05). We previously reported that both mature and semimature DCs in psoriasis lesional skin expressed high levels of *IL23A* compared to control skin (16) (**Figure 5A**). Our current study showed that the expression of *IL23A* in both mature and semimature DCs was decreased after systemic IL-17A blockade in posttreatment psoriasis lesional skin compared to pretreatment psoriasis lesional skin (**Figure 5A**, *p* < 0.05).

Reflecting the decreased DC numbers after systemic IL-17A blockade in the immunohistochemistry experiment (**Figure 2A**), both mature and semimature DC numbers were decreased in posttreatment psoriasis lesional skin compared to pretreatment psoriasis lesional skin in scRNA-seq data (**Figure 5C**). Although semimature DC cell numbers were decreased after systemic IL-17A blockade, regulatory transcriptome expression of the semimature DCs was increased after systemic IL-17A blockade – 1) The proportion of semimature DCs expressing regulatory DC markers of *BDCA-3* (*THBD*) (51) and *DCIR* (*CLEC4A*) (52) was increased in posttreatment psoriasis lesional skin compared to pretreatment psoriasis lesional skin (**Figure 5C**). 2) The expression of *CD1C* and *CD14*, which are genes of a CD1c^+^ CD14^+^ DC subset that suppresses antigen-specific T-cell responses (53, 54), was increased in posttreatment psoriasis lesional skin semimature DCs compared to pretreatment psoriasis lesional skin semimature DCs (**Figure 5B**, *p* < 0.05).

### IL-17A blockade reduces IL-17-driven inflammatory mediator expression in suprabasal keratinocytes and increases keratinocyte stem cell marker expression in basal keratinocytes in psoriasis lesional skin at single-cell cluster levels

**Figure 6:**
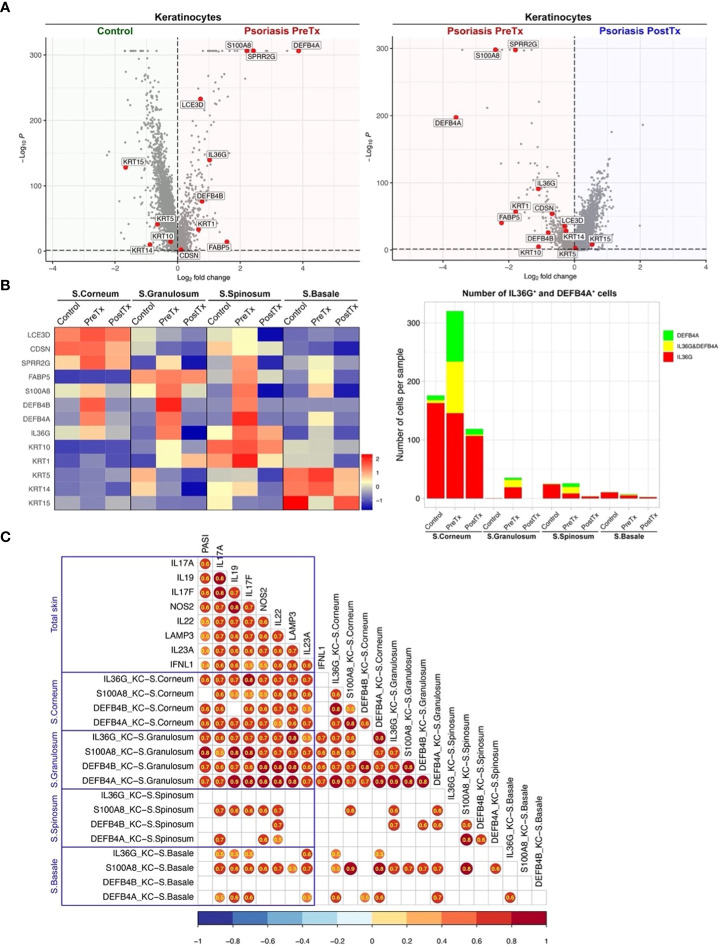
IL-17A blockade reduces IL-17-driven inflammatory mediator expression in suprabasal keratinocytes and increases keratinocyte stem cell marker expression in basal keratinocytes in psoriasis lesional skin at single-cell cluster levels. **(A)** Volcano plots displaying differentially expressed genes between control skin KCs, psoriasis lesional skin KCs before treatment (PreTx), and psoriasis lesional skin KCs after 12 weeks of systemic anti-IL-17A monoclonal antibody (secukinumab) administration (PostTx). **(B)** Heatmap illustrating the average gene expression in KC clusters representing different layers of epidermis (Stratum (S.) corneum, S. granulosum, S. spinosum, and S. basale), split by psoriasis PreTx, psoriasis PostTx, and control skin. Bar graphs displaying *IL36G* ^+^ and *DEFB4A* ^+^ cell numbers in KC clusters, split by control, psoriasis PreTx, and psoriasis PostTx. **(C)** Correlation plots displaying inter-correlations between Psoriasis Area-and-Severity Index (PASI), IL-23/T17 axis gene expression in total skin, and IL-17-driven KC inflammatory mediator expression in different epidermal layers. Circled numbers = correlation coefficients with *p* < 0.05.

We next studied how systemic IL-17A blockade modified transcriptome of KCs in different layers of epidermis. We segregated KC clusters representing different layers of the epidermis (S. corneum, S. granulosum, S. spinosum, and S. basale) and tested if psoriasis KCs express more IL-17-driven inflammatory mediators than control KCs. The expression of inflammatory mediators induced by IL-17 in keratinocytes (*IL36G*, *S100A8*, *DEFB4A*, and *DEFB4B* (3)) was increased in pretreatment psoriasis lesional skin KCs compared to control skin KCs (**Figure 6A**, *p* < 0.05). After systemic IL-17A blockade, the expression of those inflammatory mediators (*IL36G*, *S100A8*, *DEFB4A*, and *DEFB4B*) was decreased in posttreatment psoriasis lesional skin KCs compared to pretreatment psoriasis lesional skin KCs (**Figure 6A**, *p* < 0.05).

When we compared KC transcriptome in different epidermal layers, the expression of IL-17-driven inflammatory mediators (*IL36G*, *S100A8*, *DEFB4A*, and *DEFB4B*) was localized to suprabasal layers (S. corneum, S. granulosum, and S. spinosum) in pretreatment psoriasis lesional skin (**Figure 6B**). A high proportion of *IL36G* expressing KCs co-expressed *DEFB4A* in suprabasal layers (37.7% in S. corneum, 39.9% in S. granulosum and 56.7% in S. spinosum). Reflecting that inflammation in the upper layers of the epidermis determines psoriasis disease severity (erythema, thickness, and scaling components of Psoriasis Area-and-Severity Index (PASI)), KC expression of *IL36G*, *DEFB4A*, and *DEFB4B* in S. corneum and S. granulosum was correlated with PASI (**Figure 6C**, *p* < 0.05). After IL-17A blockade, KC expression of *IL36G*, *S100A8*, *DEFB4A*, and *DEFB4B* in S. corneum and S. granulosum decreased in posttreatment psoriasis lesional skin compared to pretreatment psoriasis lesional skin (**Figure 6B** and **Supplementary Table 4**, *p* < 0.05). The number of *IL36G* and/or *DEFB4A* expressing KCs in S. corneum and S. granulosum was decreased by 3 times from pretreatment psoriasis lesional skin to posttreatment psoriasis lesional skin (**Figure 6B**).

In contrast to the inflammatory mediators induced by IL-17, the expression of the KC stem cell marker (*KRT15*) was decreased in pretreatment psoriasis lesional skin KCs compared to control skin KCs (**Figure 6A**, *p* < 0.05). After systemic IL-17A blockade, the expression of *KRT15* was increased in posttreatment psoriasis lesional skin KCs compared to pretreatment psoriasis lesional skin KCs (**Figure 6A**, *p* < 0.05). The expression of *KRT15* in pretreatment psoriasis lesional skin was localized to the basal layer in pretreatment psoriasis lesional skin (**Figure 6B**). After IL-17A blockade, KC expression of *KRT15* in S. basale increased in posttreatment psoriasis lesional skin compared to pretreatment psoriasis lesional skin (**Figure 6B** and **Supplementary Table 4**, *p* < 0.05).

## Discussion

In this study, we applied multi-omics to human psoriasis skin providing a unique opportunity to compare the transcriptome of total skin (microarray and RT-PCR) and the transcriptome of skin immune cell subsets (scRNA-seq) generated from the same tissues with different techniques. When skin transcriptome changes by systemic IL-17A blockade were compared between total skin and skin immune cell subsets, IL-23/T17 axis cytokine expression was downregulated at both total skin levels and skin immune cell subset levels. The quantity of overall immune cells, including both pathogenic and regulatory subsets, was decreased in posttreatment psoriasis lesional skin compared to pretreatment psoriasis lesional skin (**Figure 2A**). Since the IL-23/T17 axis cytokine sources (T-cells, DCs, and KCs) were decreased in the predetermined volume of skin biopsy tissue (6 mm punch), total skin transcriptome of IL-23/T17 axis cytokines (*IL17A* and *IL26* from T-cells, *IL23A*, *IL12B*, and *IL20* from DCs, and *S100A8* and *IL36* from KCs) was decreased in posttreatment psoriasis lesional skin compared to pretreatment psoriasis lesional skin (**Figure 2B**).

Paired skin immune cell subset transcriptome analyses with scRNA-seq further elucidated which immune cell subsets were expressing the IL-23/T17 axis cytokines and how their transcriptome was affected by systemic IL-17A blockade. For T-cell subsets, our study showed that 1) CD161^+^ T-cell cluster was the major T17 cell cluster, but only 15.0% of CD161^+^ T-cell cluster cells were T17 cells. Single-cell clustering of immune cell subsets presented in this study was consistent with our previous single-cell paper (16), but T-cell clustering evolved as we increased the total number of single-cells for dimension reduction analyses from 23,220 to 40,026 (**Figure 3**). Our new dimension reduction and clustering analyses revealed that the majority of T17 cells in psoriasis skin were contained in the CD161^+^ T-cell cluster consistent with previous CD161^+^ T-cell studies (55) (**Figure 4C**). CD161 is a marker of all human T-cell subsets with the ability to produce IL-17, and it has been reported that IL-17-producing cells exclusively originate from naïve CD161^+^ T-cell precursors (56). In psoriasis, it has been reported that more CD161^+^ T-cells are present in psoriasis lesional skin compared to psoriasis nonlesional skin or normal skin (57, 58), and the greater frequency of CD161^+^ T-cells are present in prepsoriatic skin compared to normal skin, suggesting the role of CD161^+^ T-cells in the initial development of psoriatic lesions (57, 59). 2) CD161^+^ T-cell cluster was the major T-cell subset cluster expressing *CXCL13*, which is a cytokine that represents a particularly T17-specific abnormality and positively associates with psoriasis severity (44–47) (**Figure 4B**). In our single-cell dataset, 70.2% of *CXCL13*-expressing cells was T-cell cluster cells, and 73.2% of *CXCL13*-expressing T-cell cluster cells were CD161^+^ T-cell cluster cells.

For single-cell transcriptome modification of the IL-23/T17 cell axis induced by systemic IL-17A blockade, our study showed that 1) The expression of *IL23A* in dendritic cell subsets in psoriasis skin lesions was downregulated by systemic IL-17A blockade, potentially by blocking the entire feed-forward inflammatory amplification loop between T-cells, DCs, and KCs (**Figure 5A**), 2) Systemic IL-17A blockade removed 100% of *IL17A*-expressing T-cells. Although anti-IL-17A monoclonal antibody does not directly target IL-17F, systemic IL-17A blockade also removed 95% of *IL17F*-expressing T-cells in psoriasis skin, potentially mediated by IL-23 reduction in dendritic cells (**Figure 4C**). 2) The expression of IL-17-driven inflammatory mediators (*IL36G*, *S100A8*, *DEFB4A*, and *DEFB4B*) in KCs was localized to suprabasal KCs (**Figure 6B**). The expression of those IL-17-driven inflammatory mediators in suprabasal KCs was correlated with psoriasis severity (**Figure 6C**) and was downregulated by systemic IL-17A blockade (**Figure 6B**).

Mapping regulatory DC transcriptome is challenging because a master regulator of regulatory DCs is not identified. All regulatory DC markers of *BDCA-3* (*THBD*) (51), *DCIR* (*CLEC4A*) (52), *LILRB1* (60), *LILRB2* (*ILT4*) (61), *LILRB4* (*ILT3*) (61) and combination of *CD1C* and *CD14* (53, 54) are surface markers that are not specific for DCs. Since the markers are expressed in other skin cells, total skin transcriptome changes of those markers did not represent transcriptome changes of regulatory DCs (**Figure 2B**). Instead, skin immune cell subset transcriptome analyses of the semimature DC cluster, which we previously reported as regulatory DCs in psoriasis skin (16), showed regulatory transcriptome promotion by systemic IL-17A blockade in regulatory DCs – 1) The proportion of semimature DCs expressing regulatory DC markers of *BDCA-3* (*THBD*) (51) and *DCIR* (*CLEC4A*) (52) was increased in posttreatment psoriasis lesional skin compared to pretreatment psoriasis lesional skin (**Figure 5C**). 2) The expression of *CD1C* and *CD14* [markers of CD1c^+^ CD14^+^ DC subset that suppresses antigen-specific T-cell responses (53, 54)] was increased in posttreatment psoriasis lesional skin semimature DCs compared to pretreatment psoriasis lesional skin semimature DCs (**Figure 5B**, *p* < 0.05).

Clinically, when moderate-to-severe psoriasis patients were treated with systemic anti-IL-17A monoclonal antibody (secukinumab) administration for 52 weeks and then stopped the treatment, 16.0% of them maintained response after treatment withdrawal over the next 52 weeks without need for retreatment (12, 13). Our study may explain how those patients restored immune tolerance to psoriasis autoantigens that may have prevented recurrence of psoriasis off the treatment. In addition to the co-modulation of IL-17A and IL-17F by direct and indirect effects on T-cells including IL-23 reduction in dendritic cells, systemic IL-17A blockade may have effectively modified regulatory transcriptome of regulatory DCs (**Figures 5B**, **6C**) in those patients.

Our study has limitations: 1) Our single-cell analysis approach relied on immune cell emigration from the skin biopsies in culture. Whilst this is a recognized approach to isolate immune cells from the skin (16), some skin resident immune cells may not be migratory and may have been excluded in the immune cell subset analyses. 2) The unique challenges of scRNA-seq data analysis, including the sparsity of the scRNA-seq dataset and the dropout of lowly expressed genes, are widely acknowledged (62–67). In particular, differentially expressed gene analyses of scRNA-seq data are biased towards highly expressed genes (68), while the target genes investigated in the study (T17 axis cytokines) are lowly expressed genes in specific T-cell or DC subsets (16). To maximize the recovery of differentially expressed genes, we used a Wilcoxon rank sum test at total immune cell (T-cell, DC, or KC) levels and a spatial autocorrelation analysis (24, 25) at immune cell subset levels. However, we observed the discrepancies of IL23R status between the average expression visualized in the heatmap (**Figure 4B**) and the spatial autocorrelation test results (**Supplementary Table 3**) in Treg cluster analyses. The average expression of IL23R was higher in posttreatment Tregs compared to pretreatment Tregs (**Figure 4B**) but the difference was not statistically significant in the spatial autocorrelation test. 3) The transcriptome analyses were not validated by functional studies at protein levels. 4) Different body sites can harbor different microbes and may have different immune cell compositions, but the location of skin biopsies was not matched between comparison groups (Skin biopsy location of each sample is listed in **Supplementary Table 1**).

## Conclusion

Our study provides an early demonstration that systemic anti-IL-17A monoclonal antibody administration blocks the entire IL-23/T17 cell axis depleting or modulating 100% of *IL17A* ^+^ T17 cells and 95% of *IL17F* ^+^ T17 cells at single-cell levels (**Supplementary Figure 3**). In addition, systemic anti-IL-17A monoclonal antibody administration promotes regulatory DCs, while strongly downregulating the expression of *IL23A* in dendritic cells in psoriasis skin lesions. Thus, we hypothesize that a monoclonal blockade of pathogenic T-cells, such as an IL-17A blockade or an IL-23p19 blockade, may induce expansion of regulatory immune cells subsets or expression of cytokines involved in skin homeostasis (9, 10, 69). To further test the hypothesis, we are currently conducting a clinical trial of short-term anti-IL-23p19 monoclonal antibody administration to induce long-term disease remission that incorporates scRNA-seq analyses of human skin before/after the monoclonal antibody administration (NCT04630652). We are hoping to better understand the regulatory immune cell promotion by blocking the IL-23/T17 cell axis and develop personalized medicine approaches to cure psoriasis without recurrence.

## Data availability statement

The datasets presented in this study can be found in online repositories. The names of the repository/repositories and accession number(s) can be found below: GSE183047 and GSE226244 (GEO).

## Ethics statement

The study protocol and informed consent were approved by the Institutional Review Board of the Rockefeller University, New York, NY, USA. The study was conducted in accordance with Good Clinical Practice and the Declaration of Helsinki, and all subjects provided written informed consent before entering the study. The studies were conducted in accordance with the local legislation and institutional requirements. The participants provided their written informed consent to participate in this study.

## Author contributions

All the authors have made substantial contributions to the acquisition, analysis, or interpretation of the data. In addition, all the authors have contributed to the drafting or editing of the manuscript and gave their approval for the version submitted. JK and JGK designed the clinical trial, experiments, and laboratory protocols. JK, NK, IC, KK, and VC contributed to patient consent and care. JL and XL conducted single-cell and microarray studies. DR conducted immunohistochemistry studies. JK, YK, SG, WZ, JC, and JGK interpreted the data. JK and JGK verified all the underlying data. All authors contributed to the article and approved the submitted version.

## Supplementary material

Supplementary Figure 1 The Uniform Manifold Approximation and Projection plot (UMAP) of the initial non-linear dimension reduction and clustering analysis **(A)** and the final clusters for the downstream differential expression testing **(B)**.

Supplementary Figure 2 Alluvial diagram visualizing how individual immune cells in single-cell RNA sequencing data are allocated across categorical dimensions. Left column: Patients -10 psoriasis patients and 10 control healthy volunteers. Middle column: Treatment - psoriasis pretreatment (Psoriasis_preTx), psoriasis posttreatment (Psoriasis_postTx) after weeks 12, 24, and 48 of systemic anti-IL-17A administration, and control, Right column: immune cell clusters – NK cell, CD161 T-cell, CD8 T-cell, CD4 T-cell, regulatory T-cell (Treg), mature dendritic cell (DC), semimature DC, melanocyte, Keratinocyte in S (Stratum) corneum, S. granulosum, S. spinosum, S. basale, and fibroblast clusters.

Supplementary Figure 3 A summary of different transcriptomic impacts of anti-IL-17A blockade on human psoriasis skin at the levels of total skin and clusters of T-cell, dendritic cell, and keratinocyte subsets. DC = dendritic cell, KC = keratinocyte.
