# Supplementary figures and images for: Multi-omics segregate different transcriptomic impacts of anti-IL-17A blockade on type 17 T-cells and regulatory immune cells in psoriasis skin
Source: Front Immunol. 2023 Sep 12;14:1250504. doi: 10.3389/fimmu.2023.1250504 (PMC10536146; doi:10.3389/fimmu.2023.1250504)

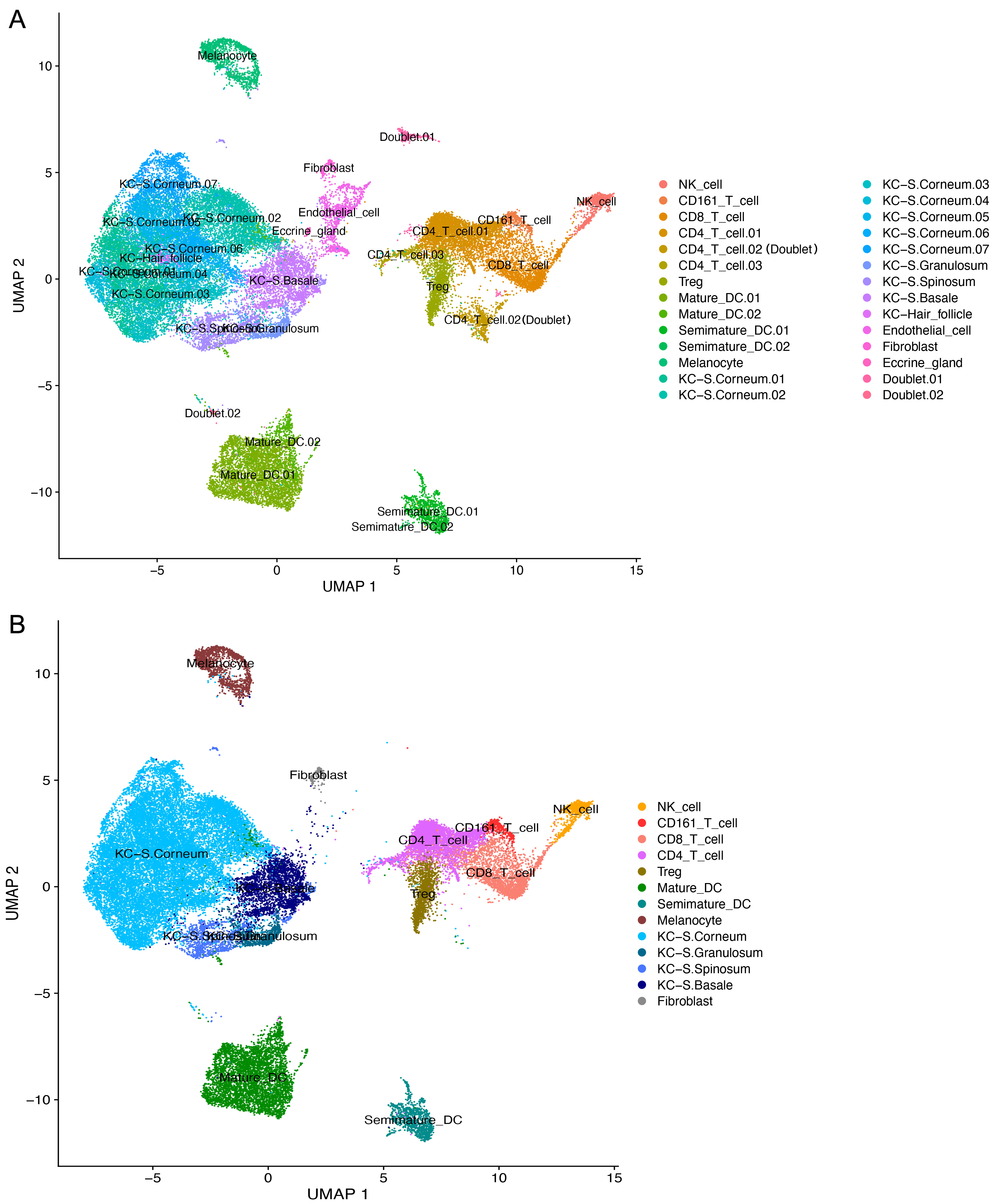

Supplement: Supplementary Figure 1 — The Uniform Manifold Approximation and Projection plot (UMAP) of the initial non-linear dimension reduction and clustering analysis (A) and the final clusters for the downstream differential expression testing (B). [file Image_1.jpg]

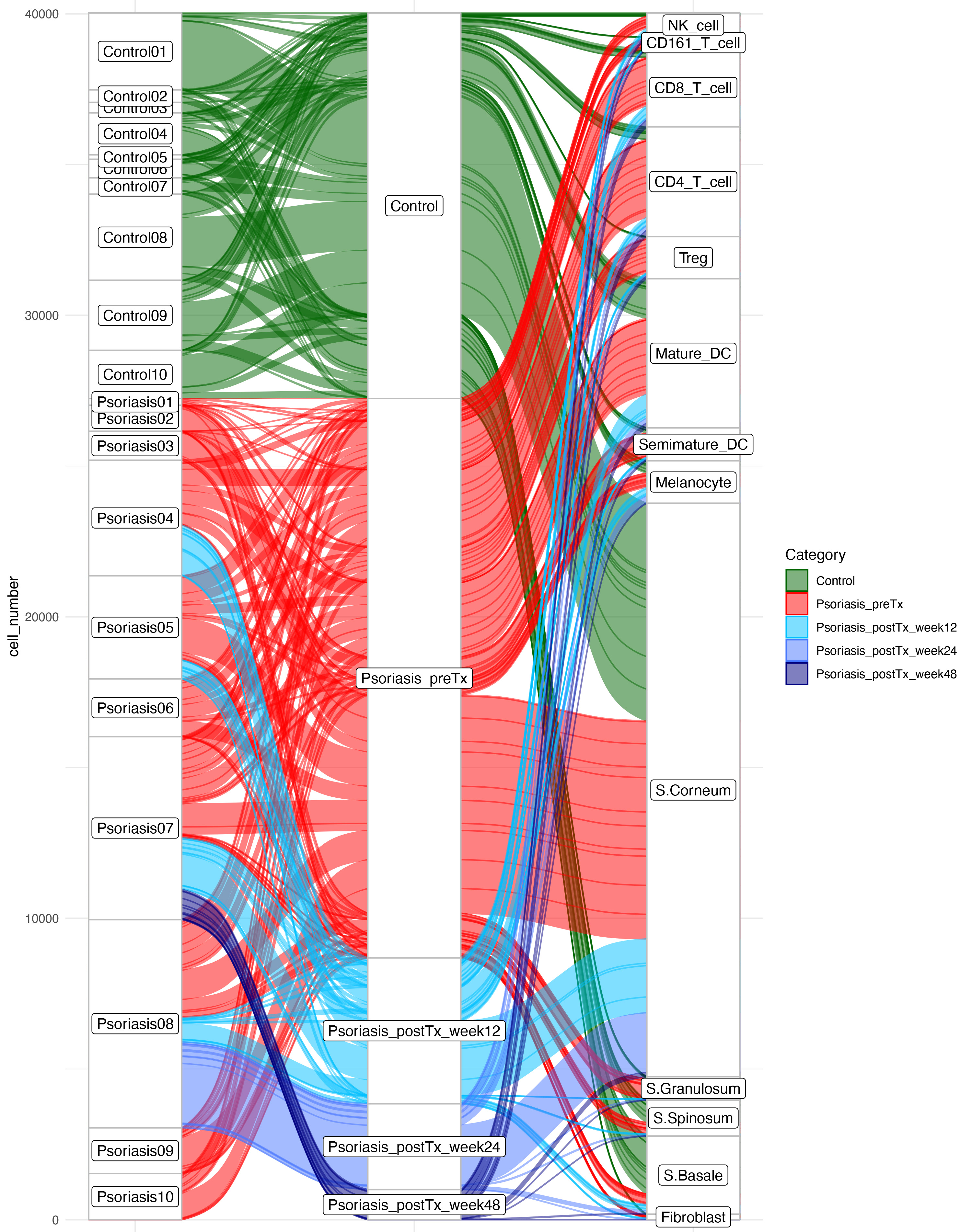

Supplement: Supplementary Figure 2 — Alluvial diagram visualizing how individual immune cells in single-cell RNA sequencing data are allocated across categorical dimensions. Left column: Patients -10 psoriasis patients and 10 control healthy volunteers. Middle column: Treatment - psoriasis pretreatment (Psoriasis_preTx), psoriasis posttreatment (Psoriasis_postTx) after weeks 12, 24, and 48 of systemic anti-IL-17A administration, and control, Right column: immune cell clusters – NK cell, CD161 T-cell, CD8 T-cell, CD4 T-cell, regulatory T-cell (Treg), mature dendritic cell (DC), semimature DC, melanocyte, Keratinocyte in S (Stratum) corneum, S. granulosum, S. spinosum, S. basale, and fibroblast clusters. [file Image_2.jpg]

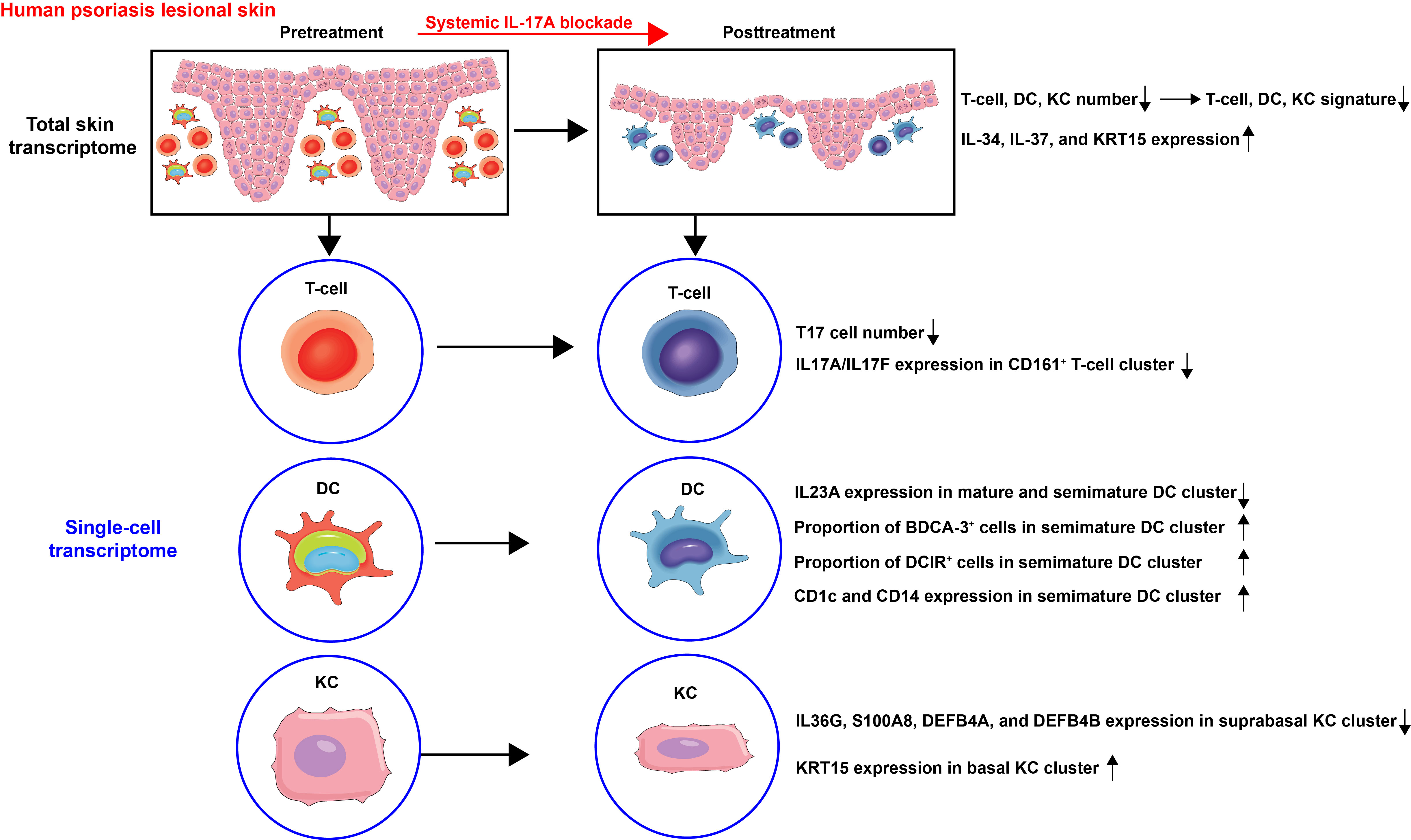

Supplement: Supplementary Figure 3 — A summary of different transcriptomic impacts of anti-IL-17A blockade on human psoriasis skin at the levels of total skin and clusters of T-cell, dendritic cell, and keratinocyte subsets. DC = dendritic cell, KC = keratinocyte. [file Image_3.jpg]
