# Supplementary material for: Multi-omics segregate different transcriptomic impacts of anti-IL-17A blockade on type 17 T-cells and regulatory immune cells in psoriasis skin
Source: Front Immunol. 2023 Sep 12;14:1250504. doi: 10.3389/fimmu.2023.1250504 (PMC10536146; doi:10.3389/fimmu.2023.1250504)
Supplement: Supplementary file 5 [file Table_2.docx]

**Supplemental Table 2. Primers used for RT-PCR**

| **Gene Symbol(s)** | **Gene Name(s)** | **Assay ID** |
| --- | --- | --- |
| RPLP0 | ribosomal protein lateral stalk subunit P0 | Hs00420895_gH |
| NOS2 | nitric oxide synthase 2 | Hs01075529_m1 |
| IL17A | interleukin 17A | Hs00174383_m1 |
| IFNG | interferon gamma | Hs00989291_m1 |
| CD274 | CD274 molecule | Hs01125301_m1 |
| CTLA4 | cytotoxic T-lymphocyte associated protein 4 | Hs03044418_m1 |
| FOXP3 | forkhead box P3 | Hs01085834_m1 |
| IDO1 | indoleamine 2,3-dioxygenase 1 | Hs00984148_m1 |
| LAMP3 | lysosomal associated membrane protein 3 | Hs00180880_m1 |
| KRT16 | keratin 16 | Hs00955082_g1 |
| IL12B | interleukin 12B | Hs01011518_m1 |
| IL19 | interleukin 19 | Hs00604657_m1 |
| IL20 | interleukin 20 | Hs00218888_m1 |
| IL23A | interleukin 23 subunit alpha | Hs00900828_g1 |
| IL24 | interleukin 24 | Hs01114274_m1 |
| IL26 | interleukin 26 | Hs00218189_m1 |
| CD69 | CD69 molecule | Hs00934033_m1 |
| FLG | filaggrin | Hs00856927_g1 |
| IL23R | interleukin 23 receptor | Hs00332759_m1 |
| IL36G | interleukin 36, gamma | Hs00219742_m1 |
| ITGAX | integrin subunit alpha X | Hs00174217_m1 |
| S100A8 | S100 calcium binding protein A8 | Hs00374264_g1 |
| IL34 | interleukin 34 | Hs01050926_m1 |
| IL37 | interleukin 37 | Hs00367201_m1 |
| IL36RN | interleukin 36 receptor antagonist | Hs01104220_g1 |
| CD1C | CD1c molecule | Hs00233509_m1 |
| KRT15 | keratin 15 | Hs00951967_g1 |
| GZMK | granzyme K | Hs00157878_m1 |
| ITGAE | integrin subunit alpha E | Hs01025372_m1 |
| LILRB1 | leukocyte immunoglobulin like receptor B1 | Hs04401227_g1 |
| LILRB2 | leukocyte immunoglobulin like receptor B2 | Hs01629548_s1 |
| NKG7 | natural killer cell granule protein 7 | Hs01120688_g1 |
